# Evolutionary neuroeconomic adaptations of fast-spiking neurons in the human neocortex

**DOI:** 10.3389/fnsyn.2026.1741452

**Published:** 2026-01-16

**Authors:** Viktor Szegedi, Abdennour Douida, Gábor Hutóczki, László Novák, Karri Lamsa

**Affiliations:** 1 Department of Physiology, Anatomy and Neuroscience, University of Szeged, Szeged, Hungary; 2 Hungarian Centre of Excellence for Molecular Medicine (HCEMM), Szeged, Szeged, Hungary; 3 Department of Neurosurgery, University of Debrecen Clinical Centre, Debrecen, Hungary

**Keywords:** ATP - adenosine triphosphate, basket cell, fast spiking cell, *Homo sapiens*, neocortex, neuroeconomic, parvalbumin (PV)

## Abstract

*Homo sapiens* has evolved a large and complex neocortex that underlies advanced cognitive capabilities. Neural computation, however, is inherently energy-intensive, and evolutionary pressures have shaped mechanisms that optimize both computational performance and energy efficiency in the human brain. Fast-spiking interneurons, particularly basket cells, are among the most active neuron types in the neocortex, where they play a key role in coordinating time and space in the activity of neuronal networks, but their high activity levels require high metabolic resources. Because the human neocortex is significantly larger than that of rodents—and contains a higher proportion of inhibitory interneurons relative to pyramidal cells—this expansion may have created evolutionary pressure to reduce the energetic cost of fast-spiking neurons. Compared with rodents, human fast-spiking neurons exhibit adaptations that appear to lower energy expenditure while preserving rapid and precise inhibition. One such adaptation is increased input resistance, which allows both excitation and inhibition to occur with reduced transmembrane ion currents, thereby decreasing the energy required to maintain ionic gradients across the plasma membrane. Since higher input resistance also slows down membrane potential changes, these cells show secondary adaptations that maintain rapid electrical signaling. Additional modifications—such as optimized ion channel composition in soma and axon initial segment, enhanced axon myelination, simplified structure of dendritic tree, and multivesicular synapses—further improve electrical signaling and are likely to reduce metabolic demand, collectively reducing ATP consumption in the neuronal network. By integrating cellular and synaptic perspectives, this review highlights how fast-spiking neurons in the human neocortex have evolved differently from those in rodents to balance energy efficiency while maintaining computational power, providing insight into the metabolic constraints of the human brain.

## 1 Introduction

The human brain, comprising approximately 90 billion neurons, possesses the most elaborated and complex cerebral cortex—the region primarily associated with cognition, reasoning, and consciousness—among all mammals (Pakkenberg and Gundersen, 1997). Despite its remarkable volumetric expansion relative to smaller mammals, human brain neurons adhere to the same fundamental cellular signaling and metabolic principles observed across species (Azevedo et al., 2009; Herculano-Houzel, 2009). Notably, humans have about 16 billion cortical neurons, the highest number recorded among mammals, a feature thought to underlie our exceptional cognitive and behavioral capacities. In contrast, even the largest-brained species, such as cetaceans and elephants, contain fewer cortical neurons despite their greater total brain mass (Azevedo et al., 2009; Herculano-Houzel et al., 2014a; Herculano-Houzel et al., 2014b). The remarkable increase in the number of neocortical neurons and the associated expansion of the neocortex that accompany it pose metabolic and energetic challenges for the human brain (Attwell and Laughlin, 2001; Herculano-Houzel, 2011). As brain metabolism scales with size and neuron number, energetic and metabolic limitations can critically influence brain evolution, particularly in large, neuron-rich cortices like the human neocortex (Karbowski, 2007).

Neural systems have evolved under persistent pressure to balance computational capacity with limited metabolic and spatial resources. Neurons are among the most energy-demanding cell types: although the brain constitutes only about 2% of body mass, it consumes nearly 20% of the body’s oxygen (Attwell and Laughlin, 2001; Herculano-Houzel, 2009). Across mammalian species, total brain energy consumption scales with neuron number (Laughlin et al., 1998; Herculano-Houzel et al., 2011; Hyder et al., 2011). In addition, larger neurons should, in theory, impose greater metabolic costs for maintaining membrane polarization—and because energy metabolism is tightly coupled to synaptic activity (Magistretti et al., 1999)—the relatively fixed energy budget per neuron suggests that large-brained apes, particularly humans, have evolved ‘primate-specific’ strategies to optimize their neuroeconomic efficiency.

Cortical organization is dominated by excitatory neurons, typically maintaining an excitatory-to-inhibitory cell ratio of around 4:1 (Douglas and Martin, 2007; Tremblay et al., 2016; Lim et al., 2018), although this ratio varies across regions and species. In the human neocortex, the relative abundance of inhibitory interneurons is higher than in rodents, and this is highest in higher-order associative areas such as the human prefrontal cortex, possibly reflecting enhanced demands for cognitive processing (Soltesz, 2006; Kooijmans et al., 2020; Hanson et al., 2025).

Among all cortical neuron types, parvalbumin-expressing fast-spiking (PVFS) interneurons are especially energy-intensive (Kann et al., 2014). Their ability to sustain high-frequency firing depends on rapid ion fluxes across the membrane and continuous ATP-dependent Na^+^/K^+^ pump activity. Basket cells—a major subclass of PVFS interneurons—is an electrically highly active neuron type important for structuring coordinated cortical network activities (Wang and Buzsaki, 1996; Traub et al., 2001; Hasenstaub et al., 2010; Melzer et al., 2012), but this precision comes at a high metabolic cost. In the expanded human neocortex, where inhibitory neuron number and density are both increased, these energetic constraints have likely exerted evolutionary pressure to preserve speed and reliability of electrical signaling while minimizing ATP consumption.

This review explores the cellular and synaptic adaptations that help human PVFS interneurons to sustain computational performance under tight energetic constraints. Cortical microcircuit operation depends on coordinated interactions among multiple neuronal and glial cell types, all of which have likely undergone evolutionary refinement. Here, however, we focus specifically on fast-spiking interneurons, particularly those in supragranular layer 2/3, which underwent pronounced expansion during human evolution (Balaram and Kaas, 2014).

## 2 Microstructural adaptations

**Figure 1:**
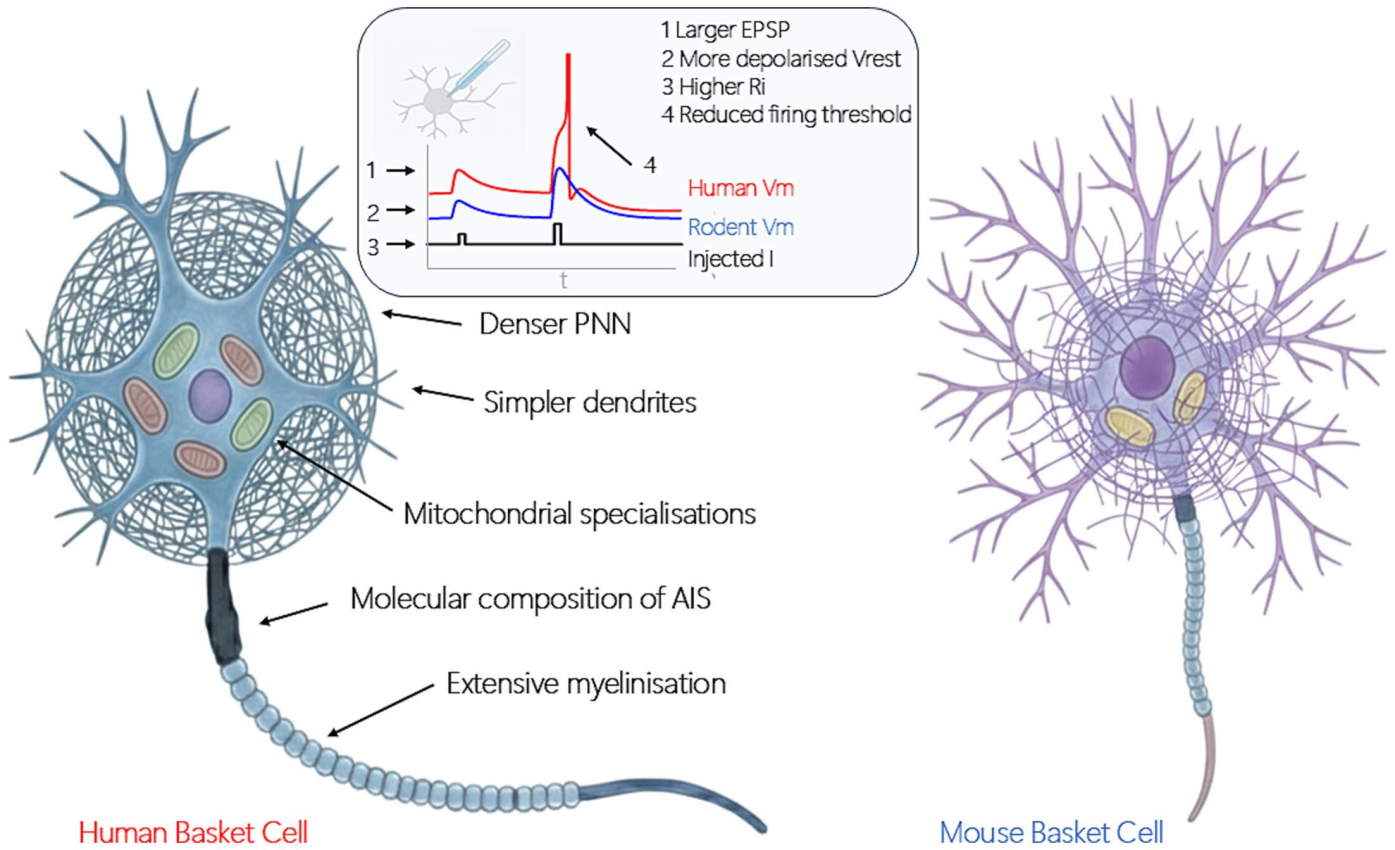
Cellular and metabolic adaptations of human parvalbumin fast-spiking (PVFS) interneurons supporting neuroeconomic efficiency. The figure illustrates some of the evolutionary specializations of human PVFS interneurons compared with their rodent counterparts. Human PVFS cells exhibit a denser perineuronal net (PNN), simplified dendritic architecture with fewer branch points, likely to have mitochondria with increased number and density with structural and metabolic adaptations, an altered molecular composition of the axon initial segment (AIS), and more extensive axonal myelination. Functionally, they show a more depolarized resting membrane potential (Vrest), higher input resistance (Ri), larger excitatory postsynaptic potentials (EPSPs), and a lower voltage threshold for action potential initiation. Together, these features enable temporally precise and energy-efficient signaling, allowing human PVFS interneurons to sustain high-frequency inhibition within the metabolically demanding human cortex.

A major challenge in scaling cortical networks of large-brained species is that larger neurons are intrinsically slower due to increased membrane capacitance and longer electrotonic path lengths (Gentet et al., 2000). Greater neurite length increases total membrane capacitance, thereby prolonging the time constant of membrane charging. It also extends the electrotonic distance over which signals must travel, leading to greater voltage attenuation and slower spread of voltage changes along neuronal processes (Das et al., 2025; Olah et al., 2025). These scaling-related factors, in theory, delay synaptic integration and action potential initiation. Yet, PVFS neurons show an evolutionarily conserved electrical phenotype with rapid and temporally precise action potential generation to synaptic input (Hu et al., 2014). This raises the question of how human PVFS neurons, which are larger and project over longer distances than those in smaller mammals, maintain the electrical rapidity within an expanded and energetically constrained cortical architecture (Benavides-Piccione et al., 2025; Kanari et al., 2025). To sustain rapid signaling despite these physical constraints, human interneurons appear to have evolved mechanisms—including specialized somatic, synaptic, and axonal properties—that maintain electrical signaling rapidity and temporal precision without substantially increasing ATP demand (Figure 1).

### 2.1 Axonal myelination

Axonal myelination is a conserved feature of PVFS interneurons across mammals. Kole et al. (2022) showed that basket cells possess myelinated axons with mitochondria clustered within myelinated segments in rodent neocortex, supporting the energetic demands of rapid signaling. Myelination reduces effective axonal membrane capacitance, approximately 0.05 μF/cm^2^ for a 10-fold myelin wrap, thereby increasing conduction velocity and decreasing the energetic cost of action potential propagation (Castelfranco and Hartline, 2015).

PVFS interneuron axons are more frequently and more extensively myelinated in humans (Micheva et al., 2018; Stedehouder et al., 2019; Benamer et al., 2020). In the mouse neocortex, roughly 25–40% of PV interneurons have detectable myelination on parts of their axons. In the human neocortex, this proportion rises to 60–80%, depending on the cortical area and age. Both human and rodent PV interneurons display proximal axonal myelination, typically beginning near the soma, distal to the AIS. Human PVFSs exhibit greater overall myelination, with longer total myelinated lengths and more internodes (Stedehouder et al., 2017).

In addition, the myelin sheath tends to be thicker in humans relative to axon diameter. The g-ratio (axon diameter/fiber diameter) is smaller in humans (~0.6–0.7) than in rodents (~0.75–0.8) for PV interneurons, indicating thicker relative myelin. The g-ratio optimizes speed. Empirically, conduction velocity is maximal around g ≈ 0.6.

The primary benefit of thicker myelin is in active saltatory conduction of the action potential along the myelinated axon from one node to the next. A thicker myelin sheet reduces cell capacitance per surface area in the axon. In addition, a thicker myelin sheet increases transmembrane resistance, since it acts as a high-resistance electrical insulator by decreasing current leakage across the membrane. Each additional layer of myelin roughly doubles the effective membrane thickness, thereby reducing membrane capacitance and increasing membrane resistance exponentially. These changes increase the membrane length constant that determines how far along an axon a passive voltage change can spread before it decays significantly. Voltage changes spread farther along the axon before decaying, and there is less voltage attenuation between nodes of Ranvier, where action potentials are generated (Table 1).

**Table 1:** Summary of differences in axonal myelinization in PVFS interneurons in the human and rodent neocortex.

Feature	Rodent PV interneurons	Human PV interneurons	Adaptation
Proportion myelinated	~25–40%	~60–80%	Greater myelination in the human cortex
Myelin onset	Begins near axon initial segment	Similar pattern	Conserved structure
Myelin thickness (g-ratio)	~0.75–0.8	~0.6–0.7	Thicker myelin in humans
Functional role	Adaptation to short-distance AP signaling	Adaptation to AP conduction over larger distances	Conserved rapid AP propagation and metabolic efficiency in human cells

Increased myelination in human cells likely reflects an adaptation to larger neuronal size and longer conduction paths in the human cortex. By lowering axonal capacitance and leak conductance, myelination enhances propagation speed and reduces energy expenditure, contributing to the metabolic efficiency of fast and precise inhibition in large cortical networks (Hartline and Colman, 2007; Fields, 2015; Benamer et al., 2020). However, if myelin is too thick, the capacitance gain saturates, and metabolic cost increases (Table 2).

**Table 2:** The effects of enhanced myelinization on electrical signal propagation and energy efficiency in human PVFS interneuron axons.

Effect of thicker myelin	Physical basis	Functional result
↑ Membrane resistance	Reduced ion leaks	Larger length constant → better electrotonic signal spread
↓ Membrane capacitance	Greater electrical insulation	Faster charging → shorter time constant
Optimized g-ratio (~0.6)	Balance of ion leak and capacitive charging	Maximal conduction velocity at g = ~0.6
↓ Metabolic cost	Fewer regenerations per unit length	Energetically efficient electrical signal transmission in the elongated axons

### 2.2 Perineuronal nets

Perineuronal nets (PNNs) are specialized extracellular matrix structures that envelop the soma and proximal dendrites of PVFS interneurons in both rodents and humans. These structures mainly contain chondroitin sulfate proteoglycans (CSPGs), hyaluronan, tenascin-R, and link proteins (see Morphett et al., 2024 for review). Yet, the PNN is more than just an extracellular scaffold: it strongly influences metabolic efficiency, ion homeostasis, and electrical signaling (for a review of PNN role in metabolism see Zhang et al., 2024).

PNN can save metabolic energy indirectly by reducing ion leak and buffering extracellular ions. First, the dense extracellular matrix limits extracellular diffusion and movement of K^+^ and Na^+^ ions near the soma, helping maintain local ionic stability, which reduces the need for constant active pumping (via Na^+^/K^+^-ATPase) - a major ATP consumer - to restore gradients after each spike. By restricting ion and water diffusion near the membrane, PNNs can lower the effective membrane capacitance of fast-spiking neurons, thereby facilitating electrical rapidity by shortening the soma time constant for membrane potential changes. In addition, PNN can reduce energy requirements by restricting cell volume changes (Tewari et al., 2018). Second, dense PNN has strengthened the electrostatic stabilization effect where negatively charged glycosaminoglycan chains in the PNN create a fixed negative potential that may help stabilize extracellular charge distribution and buffer cations, indirectly reducing leak currents and ionic perturbations during fast firing. Third, by anchoring perisomatic inhibitory synapses PNN stabilizes synaptic connections (Bosiacki et al., 2019; Fawcett et al., 2019; Auer et al., 2025). Experimental degradation of PNNs in rodents reduces firing rates and electrical gain of PVFS cells, leading to instability and impaired rhythmic activity at the neuronal network level (Hanssen et al., 2023) (Table 3).

**Table 3:** The effects of thicker and denser PNNs on the electrical properties of the soma and cell metabolic costs in human PVFS interneurons.

Function	Mechanism	Effect
Metabolic protection	Restricts ion diffusion at the extracellular site, reduces Na^+^/K^+^ pump workload, and protects from oxidative stress	↓ ATP demand during electrical activity
Electrical stability	Buffers extracellular ions, maintains local electrical field integrity	Stabilizes AP properties during repetitive activity and reduces excitability fluctuation
Signal conduction	Minimal direct effect	Slightly improves timing precision, not speed

Although the general morphology of PNNs is conserved across species, molecular composition and density appear to differ. Human and other primate PNNs show higher molecular density and more dense organization, whereas rodent PNNs are simpler and less dense (Mcgillis, 2018; Belliveau et al., 2024; da Silva et al., 2024). These molecular and structural differences may influence the biophysical properties of PNNs, and the more dense and molecularly rich PNN in humans may contribute to enhanced energy-saving function by enhanced ion buffering (Chaunsali et al., 2021) and protection against oxidate stress in human PVFS neurons (Morawski et al., 2004).

PNN also affects electrical signaling speed indirectly. Because the PNN is extracellular, it does not directly change the membrane’s capacitance or resistance. However, it can enhance the electrical signaling by stabilizing the extracellular ionic and electric field environment, which in turn affects how currents flow during synaptic and action potential activity. By reducing ion diffusion at the extracellular surface of the membrane, PNN helps maintain low extracellular K^+^ and high Na + during rapid firing. This supports consistent spike amplitude and timing, effectively increasing the temporal precision of firing.

### 2.3 K^+^ leak channels in the membrane

Human PVFS neurons exhibit higher input resistance than their rodent counterparts (Szegedi et al., 2016; Szegedi et al., 2023; Wilbers et al., 2023; Furdan et al., 2025), likely due to species-specific differences in leak channel density and transmembrane ionic conductances. Leak channels mediate passive ion fluxes that mainly determine the resting membrane potential (Gadsby, 2009) and generate an energetic cost, as ionic gradients must be continuously restored by Na^+^/K^+^-ATPases. In neurons with a larger membrane surface area, high leak conductance increases ATP demand. Thus, the higher input resistance of human PV interneurons likely reflects a reduction in passive ion leakage, lowering baseline ATP demand. The increased input resistance allows the resting membrane potential to be maintained and the generation of membrane potential changes, including synaptic excitation and inhibition and action potentials, with smaller ion currents, thereby reducing the energy cost of maintaining ionic gradients (Table 4).

**Table 4:** Effect of increased input resistance on electrical properties and energy efficiency in soma of human PVFS interneurons.

Parameter	Increased input resistance (↓ leak)	Consequence
Membrane voltage response	Larger *ΔVm* for given current	More pronounced synaptic effect
Excitability	Increases	Easier AP threshold crossing
Resting energy demand	Decreases	Fewer ions to pump back → energy saved
Temporal filtering	Slower; low-pass filtering stronger, more noise-sensitive	Slower integration, less responsiveness to fast inputs

On the other hand, higher resistance also slows the time course of membrane potential kinetics, potentially compromising temporal precision (Szegedi et al., 2023). To compensate, human PVFS neurons exhibit additional adaptations. One is the somatic expression of hyperpolarization-activated cyclic nucleotide–gated (HCN) channels, which facilitate kinetics of membrane potential changes in soma to shorten the action potential generation delay (Szegedi et al., 2023). In contrast, rodent PVFS neurons express HCN channels primarily on their axons (Roth and Hu, 2020), showing that while HCN channel expression is evolutionarily conserved, its subcellular localization has shifted in humans. This somatic localization likely enhances the rapidity to transform excitatory synaptic inputs to an AP (Kalmbach et al., 2018; Szegedi et al., 2023). HCN channels are strongly modulated by intracellular factors such as pH or Ca2+ (Combe and Gasparini, 2021), which make the electrical properties of the human PVFS cell soma a substrate for plasticity regulations.

In addition to HCN channels, inwardly rectifying potassium (Kir) channels also help regulate PVFS membrane resistance and excitability (Furdan et al., 2025). Human PVFSs achieve comparable input resistance regulation with lower Kir conductance than mouse cells, owing to their higher baseline membrane resistance. This reduced Kir activity for equal excitability regulation represents another energy-conserving adaptation, minimizing unnecessary ion flow and ATP requirements through voltage-activated ion channels (Furdan et al., 2025).

### 2.4 Dendritic tree

The electrotonic transduction of membrane potential changes is less efficient in human neurons with longer dendritic elongations. Human PVFSs in the supragranular layer, which is a particularly proliferated neocortical layer in humans compared to other mammals, have structural specializations to compensate for this (Wilbers et al., 2023). A recent study shows that human PVFSs have a simplified structure of large dendrites compared to those in rats, with a smaller number of branching points. This structural specialization improves the electrotonic propagation of membrane potential changes over longer dendritic distances, since the simplified dendritic structure with fewer branching points results in less signal attenuation. Long dendrites suffer greater voltage attenuation and temporal dispersion of signals reaching the soma. In addition, from the point of view of the main dendrite, each branch behaves as a current sink where the axial current divides, causing attenuation and slowdown of the forward propagation of voltage along the main dendrite (Wilbers et al., 2023). Signals decay faster and arrive with smaller amplitude at the soma. Therefore, PVFS interneurons in the supragranular layer of human cortex may have evolved with a simplified dendritic tree, because less division of current enables less voltage attenuation along the main shaft. The shorter total electrotonic path allows faster and stronger signal transmission from distal to proximal sites. This is particularly advantageous for fast-spiking interneurons that rely on precise temporal signaling. However, the reduced dendritic tree comes at a cost of reduced synaptic integration capacity but fewer total synapses. However, the human neocortex compensates this functional trade-off between the electrical efficiency of PVFS neurons and the network computational capacity by an increased proportional number of interneurons compared to pyramidal cells (see, e.g., Kanari et al., 2025).

In addition, fewer branches reduce metabolic cost. There is less maintenance of ion gradients across the total membrane surface area (via Na^+^/K^+^ ATPase), less metabolic maintenance of cytoplasmic and membrane-bound processes in distal locations from the soma for structural (cytoskeleton) and transport support, and reduced biosynthetic costs for ion channels and receptors (Attwell and Laughlin, 2001; Karbowski, 2007) (Table 5).

**Table 5:** The effect of dendritic length and branching on electrotonic attenuation, propagation, and metabolic cost.

Structural feature	Electrical effect	Energetic consequence	Functional trade-off
Longer dendrite	Greater attenuation, slower signal spread	Higher maintenance cost	More input area and integration
Reduced branching	Stronger, faster voltage propagation along the main shaft	Lower metabolic demand	Less synaptic integration capacity

Simplifying the architecture of dendrites by reducing the number of branch points improves electrical efficiency and reduces the energetic burden in human PVFS interneurons.

### 2.5 Axon initial segment

Compared with rodents, human parvalbumin-expressing fast-spiking interneurons (PVFSs) exhibit a lower action potential (AP) threshold at the axon initial segment (AIS) in response to somatic excitatory inputs. Together with the higher input resistance of human interneurons, this reduced AP threshold decreases the amount of depolarizing current—and therefore transmembrane ion flux—required to generate an output spike. Both features are neuroeconomically advantageous, as they minimize energetic cost per signaling event.

The reduced AP threshold in human PVFSs arises from a marked depletion of Kv1-type potassium channels in the AIS (Bakos et al., 2025). Human PVFS neurons lack Kv1.1 channels entirely, show no detectable expression of the corresponding gene *KCNA1*, and express Kv1.2 channels at substantially lower levels in the AIS compared with rodents (Bakos et al., 2025). In the absence of this Kv1-mediated outward current, smaller depolarizations are sufficient to trigger regenerative Na^+^ channel activation and initiate an AP.

Consequently, baseline K^+^ efflux at rest and near threshold is reduced during spike initiation. The accompanying reduction in both Na^+^ and K^+^ transmembrane currents lowers the workload of the Na^+^/K^+^-ATPase required to restore ionic gradients after spiking. In addition, the absence of Kv1 channels reduces the biosynthetic and maintenance costs associated with producing and trafficking these ion channels, further contributing to energetic efficiency in human PVFS interneurons.

Beyond energetic considerations, a lowered AP threshold also compensates for the slower membrane potential dynamics associated with the higher input resistance and larger somatic size of human interneurons. At the AIS, the critical transformation from excitatory postsynaptic potential (EPSP) to AP depends on how rapidly the local membrane potential reaches the Na^+^ channel activation threshold. In an AIS with low Kv1 channel density, depolarizing current originating in the soma spreads more efficiently into the AIS, while the reduced outward K^+^ conductance allows the local membrane potential to rise more steeply.

By diminishing subthreshold K^+^ “drag,” the AIS shortens the latency between EPSP onset and spike initiation. Thus, the lowered AP threshold in human PVFSs compensates for slower membrane charging, ensuring rapid and reliable action potential generation in response to excitatory synaptic input (Wilbers et al., 2023; Bakos et al., 2025) (Table 6).

**Table 6:** Reduced Kv1 channel density in the axon initial segment both lowers the energy cost of maintaining and generating action potentials and accelerates the input–output conversion process.

Outcome of reduced AIS Kv1 channels	Mechanism	Electrical and metabolic consequences
Lower AP threshold	Less outward current opposes Na^+^ influx	Easier AP initiation
Higher AIS input resistance	Reduced leak conductance	Stronger voltage response to input current
Reduced ionic fluxes per AP	Smaller Na^+^ and K^+^ gradient dissipation	Lower ATP cost per spike
Lower protein synthesis and transport demand	Protein synthesis and turnover	Reduced biosynthetic costs

## 3 Metabolic adaptations

The majority of the cell’s energy currency, ATP, is consumed by the process of restoring ionic homeostasis, primarily through Na+/K + pumps (i.e., Na/K exchangers). ATP is produced in the mitochondria, specialized organelles whose collective composition is highly dynamic and sensitive to the intracellular milieu. Human PVFS interneurons exhibit a reduced resting membrane potential, which lowers metabolic costs. Additionally, human neurons have specialized mitochondria.

### 3.1 Depolarized resting membrane potential

Human PVFSs have lower (more depolarized) resting membrane potential (Em) than their rodent counterparts (Szegedi et al., 2023), which, together with the increased input resistance and the lowered AP threshold in the cells, synergistically works to reduce the amount of excitatory current and transmembrane ion current to generate AP by excitatory synaptic inputs. This is neuroeconomical because neuronal energy consumption primarily stems from restoring electrochemical gradients through ATP-dependent ion pumps following electrical activity (Harris et al., 2012; Muangkram et al., 2023). The Na^+^/K^+^ pump accounts for up to 50% of neuronal ATP use (Attwell and Laughlin, 2001; Meyer et al., 2022).

Pump activity increases with more negative resting membrane potential, as its generation and maintenance require greater electrical work to expel Na^+^ and intake K^+^ (Gadsby et al., 1985; Hansen et al., 2002). A more depolarized resting membrane potential, as reported in human FSPV neurons (Szegedi et al., 2023; Wilbers et al., 2023), is likely to reduce this energetic burden, thus being neuroeconomical by lowering ATP demand while maintaining high excitability in human PVFSs. The depolarized Em is likely to stem from fewer open K^+^ channels, causing reduced passive K^+^ efflux, in resting conditions (for a review of two-pore-domain K + channels, or leak channels, see Lesage and Lazdunski, 2000). This also means less biosynthetic burden (Table 7).

**Table 7:** A more depolarized resting membrane potential (Em) is metabolically and biosynthetically economical because it reduces passive ionic leaks, lowers the workload of the Na^+^/K^+^-ATPase, and allows smaller K^+^ channel densities to sustain excitability.

Functional or structural outcome of more depolarized *Em*	Mechanism	Electrical and metabolic consequences
Less outward ion flux	↓ K^+^ leak conductance	↓ ATP use by Na^+^/K^+^ pump
Fewer membrane ion channel proteins	↓ Total leak channel density	↓ Biosynthetic and maintenance costs
Closer to AP firing threshold	*Em* depolarization	↑ Electrical responsiveness

### 3.2 Mitochondrial specialization

Mitochondria provide the ATP required for neuronal signaling via oxidative phosphorylation (Mattson et al., 2008). There is some evidence indicating species differences in mitochondrial structure, metabolism, and distribution between human and rodent neurons, which will be discussed in more detail below.

Comparative analyses indicate that mitochondria in the human cerebral cortex differ molecularly and functionally from those in rodents, reflecting species-specific adaptations in bioenergetics (Westi et al., 2022). These differences suggest a distinct “mitotype” in humans that supports the energetic demands of large, high-performance cortical networks (Pontzer et al., 2016). Although differences in overall brain metabolism—such as substrate usage, coupling efficiency, and mitochondrial proteome—have been reported between species, no cell-type-specific studies have yet examined these features in parvalbumin-expressing interneurons. To date, there are no direct comparative data on the number, density, or subcellular distribution of mitochondria in human versus rodent PV interneurons (Table 8).

**Table 8:** Comparative evidence for mitochondrial specializations addressing mitochondrial structure, density, and localization in human and rodent PVFS interneurons.

Mechanism	Effect
Mitochondria in PV cell axons	In mouse PV basket cells, myelinated axon internodes contain higher mitochondrial density, larger mitochondria, and increased mitochondrial volume fraction compared with unmyelinated segments (Kole et al., 2022). In humans, this has not been studied.
Human inhibitory neuron axon specializations	Human neocortex contains more myelinated inhibitory axons and these show distinctive structural/molecular features by array-tomography and EM studies (Stedehouder et al., 2017; Micheva et al., 2018). However, data comparing mitochondrial number, size, cristae density, or subcellular localization in identified human PVFS axons versus rodent PVFS axons are lacking.
Mitochondria density per neuropil volume across mammals	A multispecies ultrastructural study found conserved mitochondria density per neuropil volume across mammals, implying larger brains contain more mitochondria *per neuron* because neuron density declines with brain size (Karl et al., 2024).
Dendritic and axonal mitochondrial distribution	Rodent studies show mitochondria are enriched in dendrites and presynaptic boutons, and vary by cortical layer (Santuy et al., 2018; Faitg et al., 2021). However, cell-type-matched comparisons of subcellular mitochondrial distribution in human versus rodent PVFS interneurons are not available.
Energetics and mitochondrial function	Energy-budget analyses show that maintenance of ionic gradients and synaptic transmission dominate cortical ATP use (Attwell and Laughlin, 2001; Harris et al., 2012); mitochondria supply most neuronal ATP and are positioned to meet local energetic demand. Yet, mitochondrial coupling efficiency, proton leak, or respiration in human PVFS interneurons vs. rodent PVFS interneurons have not been studied.
PV interneuron molecular and metabolic specialization	Cell-type proteomic and transcriptomic studies in mouse reveal PV interneurons are enriched for mitochondrial and metabolic proteins, consistent with the high energy demand of fast-spiking cells (Attwell and Laughlin, 2001). Yet, there are no published, quantitative mitochondrial ultrastructure or single-cell mitochondrial physiology datasets from identified human parvalbumin PV interneurons.

In the human cortex, mitochondria display a high degree of regional and cellular specialization that presumably aligns with local computational and metabolic demands. The distribution of mitotypes follows a phylogenetic gradient, meaning that mitochondrial adaptations co-evolved with the expansion of higher-order association cortices in primate evolution (Mosharov et al., 2025). In isolated preparations, human cortical mitochondria exhibit lower oxygen consumption but equal or greater ATP output compared with mouse mitochondria, indicating more efficient oxidative phosphorylation coupling (Jeffreys and Craig, 1974). In parallel, transcriptomic and proteomic analyses reveal divergent gene expression and protein synthesis patterns between human and rodent mitochondria (for a review on cardiac cells, see Alibrandi and Lionetti, 2025), further emphasizing functional specialization at the molecular level.

Human and mouse neurons differ in mitochondrial development and metabolic activity, thereby affecting neuronal maturation. In humans, cortical neurons exhibit slower mitochondrial growth and lower oxidative metabolism, leading to a longer developmental timeline. In contrast, mouse neurons undergo faster mitochondrial maturation and generate energy more rapidly through oxidative phosphorylation (Iwata et al., 2023).

Mitotypes vary not only between species but also across neuronal classes (see Kuznetsov and Margreiter, 2009 for a review) (Fecher et al., 2019) indicating cell–type–specific energetic specializations. Even within a single neuron, the mitochondrial population is heterogeneous: axonal and dendritic mitochondria display divergent genetic and functional characteristics (Hirabayashi et al., 2024), as shown in pyramidal cells. It is therefore reasonable to assume that PVFS interneurons harbor mitochondria adapted to their exceptionally high energetic and temporal demands.

Parvalbumin basket cell myelination accumulates axonal mitochondria in internodes. A recent study in a mouse model shows that in PV-positive basket cell axons, myelinated segments show a higher mitochondrial density than unmyelinated segments within the same axon. Regarding that human PVFS cell axons are more extensively myelinated; they may also be richer in mitochondria. Given the larger size and longer axonal/dendritic distances of human cortical neurons, one might predict that human PV interneurons would have increased mitochondrial content or different localization (e.g., more mitochondria in distal processes) or enhanced coupling efficiency — but this remains to be empirically verified.

However, across many mammalian species, the density of mitochondria per unit area of neuropil in the cortex appears conserved, and mitochondria density does not scale with brain size, thus large- vs. small-brained mammals have similar mitochondria densities (Karl et al., 2024). This suggests an evolutionary constraint on mitochondrial number and density in the cortical neuropil, and also indicates that brain enlargement in mammals is accompanied by an increase in the number of mitochondria per neuron. To date, there is no direct comparison of mitochondrial number, density, or subcellular localization specifically in human PV interneurons compared to rodent PV interneurons.

## 4 Synaptic adaptations

Human cortical signaling appears optimized for energy efficiency through high-probability neurotransmitter release and sparse coding strategies (Hunt et al., 2023). In sparse coding, only a subset of neurons is active at any given moment, reducing overall metabolic load while maintaining high information capacity (Attwell and Laughlin, 2001; Willmore et al., 2011).

### 4.1 Excitatory synaptic input

Excitatory synapses from pyramidal neurons onto PV basket cells are more efficient in humans than in rodents, in terms of generating action potential discharge (Molnar et al., 2008; Komlosi et al., 2012; Szegedi et al., 2016; Szegedi et al., 2017). Human connections contain approximately four times more functional release sites—around 6.2 per active zone compared with 1.6 in rats (Molnar et al., 2016). Electron microscopy further shows that the synapses’ active zones have approximately four docked vesicles in humans, compared to one in rodents. This enables a single presynaptic spike to release more neurotransmitter and elicit a larger excitatory postsynaptic current, sufficient to recruit local interneurons synchronously (Molnar et al., 2008; Szegedi et al., 2016).

In rodents, excitatory inputs onto basket cells are more numerous (Veres et al., 2017). The higher frequency of miniature EPSPs reported in rats compared with monkeys corresponds to denser excitatory innervation (Povysheva et al., 2008). In contrast, primates achieve comparable or greater postsynaptic efficacy with fewer but stronger synapses—a distinct energy-efficient strategy.

### 4.2 Inhibitory synapses of PVFSs

Cellular reconstruction analysis reveals that human basket cells receive more GABAergic synapses on their somata and proximal dendrites than those of mice (Rebollo et al., 2025). The high number of inhibitory synapses is likely to reflect the increased number of interneurons and cell density in the human cortex compared to the rodent cortex, as evidenced by the similarity in the number of autaptic GABAergic synapses to human and mouse PV immunopositive basket cells (Szegedi et al., 2020). However, in smaller GABAA-receptor activation is needed in human PVFSs for an equal level of inhibition, measured as a relative drop of the cell input resistance by GABAAR-mediated synaptic conductance (Szegedi et al., 2020)-because of the higher starting resting input resistance of the human cells (Szegedi et al., 2023).

Human PVFSs may also produce faster and stronger inhibitory outputs compared with rodents (Wilbers et al., 2023). Individual human basket cells form more inhibitory boutons per postsynaptic pyramidal neuron than those of rodents (Karube et al., 2004; Kubota et al., 2016). The increased number of boutons per target improves transmission reliability, allowing efficient control of pyramidal neuron firing with fewer presynaptic spikes (Table 9).

**Table 9:** Comparative organization of excitatory and inhibitory synapses in human and rodent PVFS cells, with their electrophysiological and metabolic implications.

Feature	Human PVFS cells	Rodent PV FS cells	Effect on electrical signaling and metabolic cost
Excitatory input efficiency	Strong EPSPs; more likely to trigger spikes.	Weaker EPSPs; less likely to reach threshold.	Faster and more reliable recruitment of PVFSs; fewer presynaptic activations needed → lower energy per effective excitation.
Number of functional release sites per connection	~6.2 per active zone.	~1.6 per active zone.	Greater transmitter release boosts postsynaptic current and temporal precision; reduces stochastic failures, making transmission energetically efficient per spike.
Docked vesicles per active zone (EM)	~4 docked vesicles.	~1 docked vesicle.	More readily releasable vesicles → faster synaptic onset and lower jitter; high efficacy per vesicle release lowers metabolic load.
Density of excitatory inputs	Fewer but stronger excitatory synapses.	Many weak excitatory synapses.	Fewer synapses mean less maintenance; metabolically more economical with similar network performance.
Inhibitory input density (somatic and proximal dendritic)	More GABAergic synapses on soma/proximal dendrites.	Fewer inhibitory synapses.	Enables tighter control of spike timing; may increase signaling precision with moderate energetic cost for maintaining more synapses.
Autaptic GABAergic synapses	Similar number to rodents.	Similar number.	Maintains self-inhibition dynamics; neutral energy effect (similar synaptic load).
Effectiveness of inhibitory conductance	Higher resting input resistance → smaller GABA_A_R activation needed for the same inhibition.	Lower input resistance → stronger GABA_A_R activation required.	Enhanced inhibitory efficiency; less ionic flux per inhibitory event → energy-saving mechanism.
Adaptations to sparse coding	Well adapted through the above features	Less well adapted	Metabolic energy-saving mechanism at the neuronal network level.

## 5 Summary

Human parvalbumin-expressing fast-spiking interneurons combine a repertoire of structural and metabolic adaptations that sustain rapid, precise inhibition in the large human cortex. Despite their larger size and higher input resistance, they maintain fast signaling through specialized AIS and HCN channel expression, strong axonal myelination, simplified dendritic trees, and stabilizing perineuronal nets. A more depolarized resting potential and efficient mitochondrial ATP production reduce energy costs. The human PVFS interneurons are well adapted to operate within a sparse cortical coding regime, where only a limited subset of neurons is active at any given time, and which is therefore energy efficient. Their strong excitatory postsynaptic potentials, elevated intrinsic excitability, and lowered input–output transformation threshold allow them to respond rapidly to minimal excitatory drive. At the same time, their powerful and temporally precise inhibitory output ensures accurate control over local network activity, preventing runaway excitation.
